# Histopathologic Analysis of Lung Cancer Incidence Associated with Radon Exposure among Ontario Uranium Miners

**DOI:** 10.3390/ijerph15112413

**Published:** 2018-10-31

**Authors:** Avinash Ramkissoon, Garthika Navaranjan, Colin Berriault, Paul J. Villeneuve, Paul A. Demers, Minh T. Do

**Affiliations:** 1 Occupational Cancer Research Centre, Cancer Care Ontario, 525 University Avenue, Toronto, ON M5G 2L3, Canada; Avinash.Ramkissoon@cancercare.on.ca (A.R.); Garthika.Navaranjan@occupationalcancer.ca (G.N.); Colin.Berriault@occupationalcancer.ca (C.B.); PaulVilleneuve@cunet.carleton.ca (P.J.V.); Paul.Demers@cancercare.on.ca (P.A.D.); 2 Department of Health Sciences, Carleton University, 1125 Colonel by Drive, Ottawa, ON K1S 5B6, Canada; 3 Dalla Lana School of Public Health, University of Toronto, 155 College Street, Toronto, ON M5T 3M7, Canada

**Keywords:** lung cancer, histology, radon, uranium miners

## Abstract

Although radon is a well-established contributor to lung cancer mortality among uranium miners, the effects of radon decay products on different histopathologies of lung carcinoma are not well established. Using a retrospective cohort design, this study aims to examine the risks of lung cancer by histological subtypes associated with exposure to radon decay products among the Ontario Uranium Miners cohort. Cases were stratified by histological groups, and associated risks were estimated for cumulative radon exposure after adjustment for attained age and calendar period. Between 1969 and 2005, 1274 incident cases of primary lung cancer were identified. Of these, 1256 diagnoses (99%) contained information on histology. Squamous cell carcinoma was most common (31%), followed by adenocarcinoma (20%), large cells (18%), small cell lung carcinoma (14%), and other or unspecified cell types (17%). Of the histological sub-groups, small cell lung carcinoma had the strongest association with cumulative radon exposure; compared to the reference group (<1 cumulative working level months (WLM)), the highest exposure category (>60 cumulative WLM) had a relative risk (RR) of 2.76 (95% CI: 1.67–4.57). Adenocarcinoma had the lowest risk and was not significantly associated with exposure to radon decay products (RR = 1.49, 95% CI: 0.96–2.31). An increasing, linear trend in relative risk was noted with increasing cumulative WLM across small cell, squamous cell, and large cell lung carcinomas (P_trend_ < 0.05). Similarly, the excess relative risk (ERR) per WLM was highest for small cell lung carcinoma (ERR/WLM = 0.15, *p* < 0.01), followed by squamous cell carcinoma (ERR/WLM = 0.12, *p* < 0.01). Non-statistically significant excess risk was observed for adenocarcinoma (ERR/WLM = 0.004, *p* = 0.07). Our analysis of the Ontario Uranium Miners cohort data shows differences in the magnitude of the risks across four histological subtypes of lung carcinoma; the strongest association was noted for small cell lung carcinoma, followed by squamous cell, large cell, and lastly adenocarcinoma, which showed no significant associations with exposure to radon decay products.

## 1. Introduction

Radon is a well-established risk factor for lung cancer, particularly among uranium miners [1,2,3,4]. Excess risks for lung cancer mortality associated with radon exposure have been consistently demonstrated in uranium miner cohorts in the United States [5,6], France [7,8,9], Czechoslovakia [10,11,12], Germany [13,14,15], and other non-uranium mining cohorts [16,17]. Consistent with the international literature, our recent update of the Ontario Uranium Miners cohort also found an excess lung cancer risk, which persisted many years after leaving the mining industry [18].

Studies on the risk of lung cancer associated with radon exposure have historically been focused on mortality rather than incident cases. Cancer incidence is preferred because it is captured from tumour registries rather than death certificates, which has the advantage of providing more detailed information at the time of diagnosis, such as the behavior, morphology, and topology. Since not all lung cancer diagnoses resulted in mortality, incident cases provide a better estimate of the true effect of radon. From a statistical perspective, incident cases would provide a large case count, which provides more statistical power to detect an effect, if one truly exists. The additional statistical power allows for evaluation of lung cancer risks by different histologic subtypes in this study. With lung cancer morphology, there are four main histological sub-classifications: small cell carcinoma (SmCC), squamous cell carcinoma (SqCC), adenocarcinoma (AdC), and large cell carcinoma (LCC).

Histology-specific estimates of lung cancer risk are important to better understand the etiology and the role of radon decay products in lung cancer development. Globally, radon is the second leading cause of lung cancer, after smoking [19]. Among the male population in the United States, adenocarcinoma is the most common lung cancer subtype, accounting for 40% of all lung cancer cases [20]. In contrast, occupational cohorts show the most common histological subtype was squamous cell carcinoma (43%), and adenocarcinoma only accounted for 23% of all cases [21]. Differences in the proportions of histological subtypes between the general population and occupational groups implicate a different role of occupational exposures, such as radon, on the etiology of lung cancer development.

Similarly, in an earlier study of the Ontario Uranium Miners cohort, 20% of tissue samples showed adenocarcinoma, whereas the majority of the deaths were due to squamous cell and small cell carcinomas [22]. Although the lung cancer risks across lung cancer histological subtypes has not been assessed among the Ontario Uranium Miners cohort, strong associations were observed for squamous cell and small cell carcinoma among German and Czech cohorts of uranium miners [21,23]. Given that high linear energy transfer radiation is emitted during the radon decay process, it can be hypothesized that epithelial tumours (e.g., squamous cell and small cell carcinomas) would occur at a higher rate than adenocarcinoma among miners [24,25].

Mining of uranium ore in Ontario, Canada began in 1954 and continued operations until 1996, employing over 28,000 men during this period. The cohort file has recently been updated to extend the follow-up period to the end of 2007, providing more power to examine the risk of lung cancer across histological subtypes. This study presents a large cohort with detailed follow-up data and sufficient statistical power to evaluate histological-specific lung cancer risks. As such, the objective of this study is to evaluate the risk of lung carcinoma associated with exposure to short-lived radon progeny by histological subtypes among a cohort of Ontario uranium miners.

## 2. Materials and Methods

### 2.1. Study Population

This is a retrospective cohort study that includes all miners who worked at least one week in an Ontario uranium mine. Miners were excluded if they: were a probable duplicate; did not have a recorded date of birth; entered the cohort after 31 December 1996; were younger than 15 or older than 65 at first employment; had invalid dates of employment or cancer diagnosis. Very few women were employed as underground uranium miners (*n* = 413), and therefore, were also excluded from this analysis. After exclusions were applied, the final cohort consisted of 28,546 male uranium miners. A detailed description of the cohort has been published elsewhere [18,26]. Briefly, miners employed between 1954 and 1996 were identified from the Mining Master File (MMF) and National Dose Registry (NDR) [18,26,27,28,29]. The MMF contains detailed work history data for all uranium miners up until 1986, when it was discontinued. Since the MMF records stopped at 1986, the NDR was used to identify miners up to the closure of the last mine in 1996. The NDR was created by the Radiation Protection Bureau of Health Canada in 1951 to monitor workers that may be exposed to ionizing radiation. Follow-up of this cohort was conducted through record linkages to the Canadian Mortality Database for national mortality follow-up from 1954 to 2007, and to the Canadian Cancer Database for national cancer incidence follow-up from 1969 to 2005. The linkage was then enhanced using the Historic Summary Tax File. To avoid potential biases, all data linkage was conducted nationally by Statistics Canada, blinded to exposure levels and case status.

### 2.2. Estimates of Radon Progeny Exposure

In the early years of mining (1954–1957), occupational exposures to radon decay products were estimated by mine engineers using stationary area sampling [3,30]. After 1958, measurements of radon decay products were conducted by mine operators in different areas of the mines, including heading, stopes, raises, and travelways [3,27,30]. Annual radon exposure was computed based on time spent by individual miners in these different work areas and travelways, and is reported in working level months (WLM). One working level (WL) is defined as 1.3 × 105 MeV of potential energy from alpha particles per liter of air. An amount of 1 WLM is equal to an exposure to 1 WL for 170 h. All exposures were assigned to miners blinded to their lung cancer status.

### 2.3. Morphology and Histology

In Canada, each province and territory is responsible for maintaining databases of information about residents diagnosed with cancer collated from health care utilization information, pathology reports, and deaths certificates. This information is in turn collated nationally by the Canadian Cancer Registry (CCR) and maintained by Statistics Canada [31,32]. Data from the CCR is used to identify lung cancer cases for this study. This study only included incident cases where morphologic information was available; cases without morphology codes (*n* = 18) were excluded from the stratified analysis. Morphologic diagnoses were primarily confirmed using microscopic examination of tissue samples; otherwise clinical techniques were employed, including laboratory diagnostics, radiology, surgery, and endoscopy. Histologic coding was based on the International Classification of Diseases for Oncology (ICD-O-3) of lung carcinoma grouped into: (1) squamous cell carcinoma (SqCC), (2) adenocarcinomas (AdC), (3) small cell carcinoma (SmCC), (4) large cell carcinoma (LCC), and (5) other or unspecified. Table 1 provides a summary of the morphology codes used for categorizing the cases by histological subtypes.

### 2.4. Statistical Analyses

To evaluate the risk of lung carcinoma associated with exposure to short lived radon progeny by histological subtypes, complete exposure and follow-up history of the cohort was ascertained. Miners contributed person-years from the latest of their date of first employment or 1 January 1969, until the earliest of their date of death, 31 December 2005, or date of primary diagnosis of lung cancer. A cut-off age of 85 years was applied to minimize any bias caused by loss to follow-up. Person-years at risk (PYAR) were cross-classified by attained age (<55, 55–<60, 60–<65, 65–<69, 70–<75, ≥75 years), calendar period (1969–1974, 1975–1995, >1995), and cumulative radon exposure in WLM (<1, 1–10, >10–20, >20–60, >60). Categories for cumulative radon exposure, attained age, and calendar period were chosen to provide an approximately equal distribution of cases across histological groups and a sufficient sample size to provide reliable risk estimates. To account for the latency between exposure and cancer outcome, a range of lag times (0, 5, 10, 15, and 20 years) were examined. The lag time that yielded the highest risk estimates was chosen to capture the ideal exposure time window that will produce the strongest results [33].

To avoid bias caused by the healthy worker effect, internal cohort analyses were conducted using Poisson regression to estimate the relative risk (RR) of lung cancer overall, and for the four histologic subtypes of interest. Poisson regression with PROC GENMOD (SAS Version 9.4) was used to model lung cancer risk as a function of cumulative radon exposure and covariates of interest. The general form of the Poisson regression model is:

ln(I/I_0_) = β_1_X_1_ + β_2_X_2_ + … + β*_k_*X*_k_*, (1)

where X_1_, X_2_, …, X*_k_*, are independent variables, I is the incidence rate for persons with specified values X_1_, X_2_, …, X*_k_*, I_0_ is the incidence rate in the control group, and β_1_, β_2_, …, β*_k_* are the estimated regression coefficients. Exponentiation of the regression coefficients provides an estimate of the relative risk (I/I_0_), controlling for the independent variables (attained age and calendar period). Wald-based 95% confidence intervals were calculated for all risk estimates.

The χ^2^ test for linear trend was used to assess the dose response relationship between cumulative exposure to radon decay products and cancer outcomes. Given that the exposure variable in this study was categorized into five groups (<1, 1–10, >10–20, >20–60, >60 WLM), the linear test for trend was conducted by using the weighted mean dose for each category.

In order to examine cumulative radon exposure as a continuous variable, linear excess relative risk (ERR) models were used, with the general form:

RR = 1 + βX_1_, (2)

where RR is the relative risk and β is the increase in ERR per unit increase of cumulative radon exposure (WLM, X_1_). This model assumes a linear, no threshold association between cumulative radon exposure and risk of incident lung cancer [3]. Estimates of ERR/WLM with Wald-based 95% confidence intervals were modeled using the AMFIT module in EPICURE, and adjusted for attained age and calendar period.

## 3. Results

The analysis was based on a cohort of 28,546 miners. Of these, 1274 miners were identified as having a primary diagnosis of lung carcinoma. Table 2 demonstrates that on average, lung cancer cases were slightly older than non-cases (33 ± 8.9 years, *p* < 0.01) when they started mining, and worked as a miner for a longer period (5 ± 5.1 years) compared to the rest of the cohort (*p* < 0.01). 75% of the lung cancer cases occurred among the 43% of miners that were first employed before 1960 when radon levels were the highest due to poor ventilation practices (*p* < 0.01).

Table 3 shows the distribution of the lung cancer cases by histologic subtypes. Among the 1274 cases identified, 1256 had morphology codes from which the histologic group could be determined. In total, there were 391 (31%) incident cases of SqCC, 249 (20%) incident cases of AdC, 225 (18%) incident cases of LCC, and 181 (14%) incident cases of SmCC. In addition, there were 210 (17%) cases of the extracted morphological codes that did not correspond to these four major groups, such as sarcoma, which were categorized as other or unspecified. On average, SqCC and SmCC had the highest mean cumulative radon exposure (51.4 and 55.6 WLM respectively); however, this was expected since they also had the longest mean duration of employment (5.3 and 6.0 years, respectively). In contrast, AdC had the lowest mean cumulative radon exposure (37 WLM), as explained by shorter mean duration of employment (4.2 years) and starting work in later years when ventilation practices had improved. We also examined age at diagnosis by histologic subtypes (data not shown); however, there were no statistical differences across cell types.

Table 4 provides results of the Poisson regression analyses. Risk estimates presented are with no lag, 5-year lag, and 10-year lag periods. All estimates were adjusted for attained age and calendar period. The results confirm that the relative risk of lung cancer incidence remains elevated among uranium miners exposed to radon. Compared to the referent group (<1 WLM), those within the highest cumulative exposure group (>60 WLM) had a two-fold increase in risk of lung cancer (RR = 2.03, 95% CI: 1.68–2.45).

In general, increasing lag-time (from 0 to 10 years) increases the magnitude of the risk estimate, suggesting it is a more appropriate latency period. Interpretations of results are therefore based on the 10-year lag (Table 4). When compared to the referent group (<1 WLM), AdC had the lowest relative risk (RR = 1.49, 95% CI: 0.96–2.31) at the highest cumulative radon exposure (>60 WLM) among all histological subtypes. In contrast, a nearly tripled risk for SmCC was observed for the same exposure comparison (RR = 2.76, 95% CI: 1.67–4.57). Similarly, SqCC was strongly associated at the highest category of radon exposure, compared to reference (RR = 2.29, 95% CI: 1.63–3.23). The increase in cancer risks for both SmCC and SqCC were dose-dependent (*p* < 0.01) with increasing cumulative radon dose. Both LCC and the other/unspecified categories also had significant associations with radon exposure, although the magnitude of the association was smaller and the dose-response relationships were weaker.

**Figure 1:**
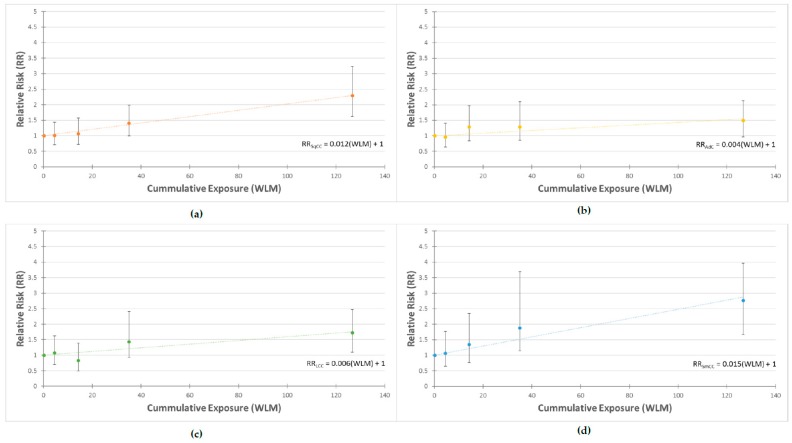
Excess relative risk by lung cancer histology groups, with 10-year lag applied and adjusted for attained age and calendar period: (**a**) Squamous cell lung carcinoma; (**b**) Adenocarcinoma; (**c**) Large cell lung carcinoma; (**d**) Small cell lung carcinoma.

The excess relative risk (ERR) was also computed to estimate the proportion of risk attributed to radon exposure, adjusting for attained age and calendar period, with 10 year lag applied. Figure 1 summarizes the results of analyses of the Ontario Uranium Miners cohort showing the ERR per WLM by histological group. The ERR/WLM was highest for SmCC (ERR/WLM = 0.15, *p* < 0.01) followed closely by SqCC (ERR/WLM = 0.12, *p* < 0.01). In contrast, non-statistically significant excess risk was observed for adenocarcinoma (ERR/WLM = 0.004, *p* = 0.07). As expected, the results of the ERR analysis reflect the findings of the relative risk analysis.

## 4. Discussion

Similar to previous studies, our analysis confirms the increased risk of lung cancer with increasing cumulative exposure to radon decay products (P_trend_ < 0.01). More importantly, this study found significantly higher risks for SqCC and SmCC than other histologic subtypes. Furthermore, the increased risk for SqCC and SmCC showed a strong dose-response relationship (P_trend_ < 0.01). Conversely, diagnosis of adenocarcinoma was not significantly associated with radon at any exposure level (P_trend_ = 0.17). This study contributes to the literature by providing empirical evidence showing lung cancer incidence associated with exposure to radon decay products for different histological subtypes among the Ontario Uranium Miners.

Our finding of elevated risk for SqCC and SmCC is consistent with previous studies and further provides empirical evidence that certain lung cell types are more radiosensitive than others [23,34]. The differences in risk estimates across histologic groups may possibly be explained by oxidative stress from oxy-radicals produced by chronic radon exposure. Over-production of oxy-radicals is known to cause pulmonary inflammation, which in turn, leads to overproduction of cytokines and chemokines [35]. In a genome-wide association study of the Saccomanno Uranium Miner cohort, a sequence variation of the interleukin-6 promoter region was found to be significantly associated with increased risk of SqCC and a shorter latency period, among radon-exposed miners [35]. This suggests that there in indeed epigenetic responses to environmental radon exposure, which may, in part, account for the differences in risk observed across histologic groups. The process of radon decay emits alpha particles which have a high linear energy transfer, but very low penetrance. Mechanistically, epithelial cells that are the front line barrier to alpha radiation would receive the majority of the dose, and would therefore be at highest risk of tumorigenesis. These findings suggest that the radon decay process can more readily initiate DNA damage of epithelial tissues, such as squamous and small cell carcinomas through direct irradiation compared to adenocarcinoma [25].

Studies of residential radon have previously quantified the association between radon exposure and histologic subtypes of lung cancer. Barros-Dios et al. (2012) found an elevated risk of lung cancer with radon exposure across all histologic subtypes [36]. Similarly, they found the highest risk associated with other cell types and SmCC, but contrary to our findings, the elevated risk in SqCC was non-significant. That study, however, was focused on lower concentrations of radon exposure in residential settings, which may account for some of the differences between our findings. A recent systematic review also found that exposure to residential radon had the strongest association with SmCC compared to any other histological group among the general population and miners, which is consistent with our findings [37].

A major limitation of these analyses is the lack of individual level smoking information for this cohort, as it is the leading cause of lung cancer. Studies of lung cancer often adjust for smoking status, and it has been found to have synergistic interactions with radon exposure, underscoring its importance in studies of lung cancer risk [21]. The risks for each histologic group follow a similar pattern to the risks associated with smoking, which may be partially explained by the interaction between radon exposure and smoking status. Unfortunately, this hypothesis cannot be tested within the Ontario Uranium Miners cohort. Although limited by a lack of smoking data, the study has significant strengths including its quantitative exposure assessment, access to national tumor registry data, and large sample size, which facilitated our investigation of the dose-response relationship across the four major histological subtypes of lung cancer.

An issue with using a single lag time is that the latency period for lung cancer can range from 5 to 20 years, which is based on a number of factors, perhaps including the histologic group. Using a single lag time may not provide enough information, as this analysis demonstrates that some histologic groups have peak risk with 5-year lag, and others with 10-year lag. The analyses also did not consider the effect of sex on the risk estimates. Because there were so few female uranium miners in Ontario (*n* = 413), they were excluded from the risk analysis in this study.

Another strength of this study is that incident cases are used rather than deaths. Because tumour registry data is much more detailed than death certificates, using incident cases permits analysis by histologic group. Furthermore, incident cases are not influenced by changes in cancer treatment practices over time that can impact survival duration following initial diagnosis. In this study, the national Canadian Cancer Registry linkage contained ICD-O-3 morphology coding, allowing for analysis of incident cases. From the morphology coding, the incident cases may be grouped by histology for the stratified analysis, making our cohort amendable to analyses similar to the German cohort [21,38].

Historically, most studies have examined lung cancer mortality, where differences across histologic groups cannot be examined due to the lack of information on tumour morphology on death certificates. The lack of cancer registries with appropriate morphology data in other jurisdictions, more recent and shorter follow-up periods, and insufficient statistical power may also be reason for sparse literature on stratified analyses by histological subtype.

Future work with these data includes investigating the inverse dose rate effect in this cohort, stratified by histologic subtype; the same cumulative dose protracted over a longer time may result in certain histologic subtypes more frequently than others, and further investigation is warranted. This analysis also demonstrates the capacity for histologic analyses for other incident cancers; however, the issue of statistical power may limit the ability to stratify less common cancers.

## 5. Conclusions

This is the first comprehensive study of lung cancer risk associated with exposure to radon decay products evaluated by histological subtypes among a cohort of Ontario Uranium Miners. Based on our analysis of the Ontario Uranium Miners cohort file, we found that the incidence of lung cancer was strongly associated with occupational radon exposure. Within this cohort, the highest risk was observed for small cell lung carcinomas, followed by squamous cell lung carcinomas, large cell lung carcinomas, and lastly adenocarcinoma. While the results of our study is limited by the lack of smoking information, the excess risks observed for the different histological subtypes for our study is in agreement with the literature. Findings from this study contribute to the growing body of empirical evidence on lung carcinoma associated with exposure to short-lived radon progeny by histological subtypes.

## Figures and Tables

**Table 1:** Classification of incident lung cancers by histology.

Lung Cancer Histology	ICD-O-3 Morphology Codes
Squamous cells (SqCC)	8050–8053, 8060, 8070–8078, 8083–8084
Adenocarcinoma (AdC)	8140, 8211, 8230–8231, 8250–8260, 8323, 8480–8481, 8490, 8550–8551, 8570–8574, 8576
Large cells (LCC)	8010–8012, 8014, 8015, 8020, 8021, 8022, 8030, 8031, 8035, 8310, 8046
Small cells (SmCC)	8041–8045
Other or Unspecified	Other specified carcinoma (8246), sarcoma (8800–8811, 8830, 8840–8921, 8990–8991, 9040–9044, 9120–9133, 9150, 9540–9581), unspecified (8000–8005)

**Table 2:** Characteristics of the Ontario Uranium Miners cohort.

Characteristics	Cases *n* = 1274	Non-Cases *n* = 27,272	Test for Difference *p*-Value
Age at entry into study (Years)			
Median	32	26	<0.0001
Mean ± SD	32.85 ± 8.88	28.57 ± 8.53	
Range	17–65	16–65	
Total Duration of Employment (Years)			
Median	3.0	2.5	<0.0001
Mean ± SD	5.04 ± 5.10	4.01 ± 4.05	
Range	0.5–31.0	0.5–32.0	
Cumulative radon exposure (WLM)			
Median	18.89	6.03	<0.0001
Mean ± SD	45.86 ± 70.33	23.06 ± 45.06	
Range	0–875.13	0–800.00	
Birth year, *n* (%)			
<1900	0 (0%)	26 (0.10%)	<0.0001
1900–1919	198 (15.54%)	2104 (7.71%)	
1920–1939	950 (74.57%)	12,081 (44.30%)	
1940–1959	125 (9.81%)	11,314 (41.49%)	
≥1960	1 (0.08%)	1747 (6.41%)	
Year first employed, *n* (%)			
<1960	956 (75.04%)	11,780 (43.19%)	<0.0001
1960–1969	107 (8.40%)	2426 (8.90%)	
1970–1979	157 (12.32%)	8496 (31.15%)	
1980–1989	52 (4.08%)	4475 (16.41%)	
≥1990	2 (0.16%)	95 (0.35%)	

WLM = Working Level Months. *p*-value < 0.05 indicates a significant difference between cases and non-cases.

**Table 3:** Characteristics of lung cancer diagnosis by histology subtypes, Ontario uranium miners.

Characteristics	Squamous Cells (*n* = 391)	Adenocarcinoma (*n* = 249)	Large Cells (*n* = 225)	Small Cells (*n* = 181)	Other/Unspecified (*n* = 210)
Age at entry into study (years)					
Median	32	29	33	30	34
Mean ± SD	32.9 ± 8.4	30.8 ± 8.6	33.7 ± 9.3	31.8 ± 8.5	35.2 ± 9.3
Range	17–61	17–62	17–63	19–61	17–65
Total Duration of Employment (years)					
Median	3.25	3	3	4	3
Mean ± SD	5.3 ± 5.2	4.2 ± 4.2	4.9 ± 4.7	6.0 ± 6.2	5.1 ± 5.2
Range	0.5–30	0.5–26.5	0.5–31	0.5–29	0.5–25.5
Cumulative radon exposure (WLM)					
Median	22.6	14.9	16.3	23.1	18.6
Mean ± SD	51.4 ± 75.3	37.0 ± 61.2	41.9 ± 57.2	55.7 ± 77.4	43.3 ± 78.0
Range	0–759.2	0–579.0	0–267.3	0–355.9	0–875.1
Birth year, *n* (%)					
<1900	0 (0%)	0 (0%)	0 (0%)	0 (0%)	0 (0%)
1900–1919	60 (15.35%)	22 (8.84%)	41 (18.22%)	22 (12.15%)	48 (22.86%)
1920–1939	305 (78.01%)	192 (77.11%)	155 (68.89%)	138 (76.24%)	147 (70.00%)
1940–1959	26 (6.65%)	34 (13.65%)	29 (12.89%)	21 (11.60%)	15 (7.14%)
≥1960	0 (0%)	1 (0.40%)	0 (0%)	0 (0%)	0 (0%)
Year first employed, *n* (%)					
<1960	306 (78.26%)	180 (72.29%)	156 (69.33%)	136 (75.14%)	160 (76.19%)
1960–1969	32 (8.18%)	20 (8.03%)	20 (8.89%)	16 (8.84%)	19 (9.05%)
1970–1979	39 (9.97%)	35 (14.06%)	39 (17.33%)	23 (12.71%)	21 (10.00%)
1980–1989	14 (3.58%)	12 (4.82%)	10 (4.44%)	6 (3.31%)	10 (4.76%)
≥1990	0 (0%)	2 (0.80%)	0 (0%)	0 (0%)	0 (0%)

WLM = Working Level Months. Percentages may not add up to exactly 100 percent due to rounding error.

**Table 4:** Risks associated with cumulative exposure to radon progeny in working level months by lung cancer histology.

Cumulative Exposure to Radon Progeny (WLM)	No Lag			5 Year Lag			10 Year Lag		
	Cases (*n*)	Relative Risk * (95% CI)	Test for Linear Trend *p*-Value	Cases (*n*)	Relative Risk * (95% CI)	Test for Linear Trend *p*-Value	Cases (*n*)	Relative Risk * (95% CI)	Test for Linear Trend *p*-Value
All Lung Cancers									
<1	188	Reference	<0.0001	188	Reference	<0.0001	197	Reference	<0.0001
1–10	290	0.91 (0.75, 1.09)		290	1.00 (0.83, 1.21)		286	1.05 (0.87, 1.26)	
10–20	185	1.03 (0.84, 1.27)		186	1.13 (0.92, 1.39)		183	1.17 (0.95, 1.44)	
20–60	313	1.30 (1.08, 1.57)		312	1.40 (1.16, 1.68)		313	1.46 (1.21, 1.76)	
>60	298	1.84 (1.52, 2.22)		298	1.98 (1.64, 2.40)		295	2.03 (1.68, 2.45)	
Squamous cells									
<1	49	Reference	<0.0001	49	Reference	<0.0001	54	Reference	<0.0001
1–10	87	0.99 (0.69, 1.40)		87	1.03 (0.72, 1.47)		83	1.01 (0.71, 1.43)	
10–20	52	1.01 (0.68, 1.50)		52	1.06 (0.71, 1.57)		52	1.06 (0.72, 1.57)	
20–60	97	1.37 (0.96, 1.94)		97	1.42 (1.00, 2.02)		96	1.40 (0.99, 1.98)	
>60	106	2.20 (1.55, 3.12)		106	2.29 (1.62, 3.25)		106	2.29 (1.63, 3.23)	
Adenocarcinoma									
<1	44	Reference	0.2168	44	Reference	0.2471	45	Reference	0.1736
1–10	61	0.88 (0.59, 1.30)		61	0.99 (0.67, 1.46)		61	0.95 (0.64, 1.41)	
10–20	44	1.18 (0.77, 1.81)		44	1.26 (0.82, 1.95)		43	1.28 (0.84, 1.97)	
20–60	56	1.18 (0.78, 1.77)		56	1.28 (0.85, 1.92)		56	1.28 (0.85, 1.92)	
>60	44	1.37 (0.89, 2.13)		44	1.49 (0.96, 2.31)		44	1.49 (0.96, 2.31)	
Large cells									
<1	36	Reference	0.0088	36	Reference	0.0087	39	Reference	0.0148
1–10	56	0.92 (0.60, 1.40)		56	1.03 (0.67, 1.57)		55	1.07 (0.7, 1.62)	
10–20	27	0.79 (0.48, 1.31)		27	0.88 (0.53, 1.46)		26	0.83 (0.5, 1.39)	
20–60	55	1.20 (0.78, 1.86)		55	1.32 (0.85, 2.04)		56	1.43 (0.93, 2.19)	
>60	51	1.68 (1.08, 2.62)		51	1.84 (1.18, 2.88)		49	1.72 (1.09, 2.69)	
Small cells									
<1	28	Reference	<0.0001	28	Reference	<0.0001	28	Reference	<0.0001
1–10	34	0.77 (0.46, 1.27)		34	0.87 (0.52, 1.44)		35	1.07 (0.64, 1.77)	
10–20	26	1.10 (0.64, 1.90)		27	1.28 (0.74, 2.20)		26	1.34 (0.77, 2.35)	
20–60	47	1.52 (0.94, 2.47)		46	1.64 (1.00, 2.69)		46	1.88 (1.15, 3.09)	
>60	46	2.28 (1.39, 3.73)		46	2.50 (1.52, 4.12)		46	2.76 (1.67, 4.57)	
Other/ Unspecified									
<1	29	Reference	0.0339	29	Reference	0.0323	29	Reference	0.0248
1–10	45	0.85 (0.53, 1.36)		45	0.94 (0.58, 1.50)		45	1.05 (0.65, 1.69)	
10–20	36	1.16 (0.71, 1.91)		36	1.30 (0.79, 2.14)		36	1.51 (0.92, 2.49)	
20–60	51	1.19 (0.74, 1.89)		51	1.25 (0.78, 2.01)		52	1.34 (0.83, 2.17)	
>60	49	1.65 (1.03, 2.65)		49	1.78 (1.10, 2.87)		48	1.93 (1.19, 3.13)	

* Relative risk estimates are adjusted for attained age and calendar period; WLM = Working Level Months; CI = Confidence Interval; *p*-value < 0.05 indicates a positive trend in relative risk as cumulative WLM increases.
